# Prevalence of peripheral arterial disease and associated factors among hypertensive patients attending two tertiary hospitals of Addis Ababa, Ethiopia

**DOI:** 10.1186/s12872-025-05451-8

**Published:** 2025-12-18

**Authors:** Semira Khairdin Jemal, Subah Abderehim Yesuf

**Affiliations:** 1 Department of Nursing, School of Nursing and Midwifery, College of Health Sciences, Addis Ababa University, Addis Ababa, Ethiopia; 2 Department of Family Medicine, St. Paul’s Hospital Millennium Medical College, Addis Ababa, Ethiopia

**Keywords:** Hypertension, Prevalence, Peripheral artery disease, Associated factors, Ethiopia

## Abstract

**Background:**

Peripheral arterial disease (PAD) is a common clinical condition among hypertensive patients, with potentially severe complications such as coronary heart disease, stroke and foot gangrene. There is scarcity of empirical data regarding the magnitude and risk factors of peripheral arterial disease among hypertensive patients in sub-Saharan Africa, including Ethiopia.

**Objective:**

To assess the prevalence of peripheral artery disease and its associated factors among patients with hypertension attending two tertiary hospitals of Addis Ababa, Ethiopia, 2023.

**Methods:**

A facility-based, cross-sectional study was conducted at Tikur Anbesa Specialized Hospital and St. Paul’s Hospital Millennium Medical College. Convenience sampling technique was adopted to recruit patients with hypertension having follow-up at both of selected tertiary hospitals. Data were collected using an interviewer-administered, pretested locally translated version of the questionnaire. The collected data were entered into Epi-info 7.2.2.2, and then analyzed using SPSS version 26. Descriptive analysis such as median + interquartile range, and percentages were used to summarize the background characteristics. Binary and multiple logistic regression were employed to determine factors associated with peripheral artery disease. Odds ratios with 95% confidence intervals were computed, and a p-value < 0.05 was considered significant. Texts, tables and figures were used to present results.

**Results:**

A total of 408 patients with hypertension were included in this study, making a response rate of 96.7%. Patients’ median age (interquartile range) was 60 (54–67) years, with female predominance (56.9%; *n* = 232). The overall magnitude of peripheral arterial disease was 31.4% (95%CI: 26.85%–35.89%). History of cigarette smoking [(AOR = 9.99; 95%CI: (4.22,23.66)], suboptimal blood pressure control [(AOR(95%CI): 2.73(1.65,4.51)], positive family history of PAD [(AOR(95%CI) = 3.59(1.37,9.42)], and history of leg pain [AOR = 1.98 (95%CI: (1.23,3.17)] were significantly associated with the disease.

**Conclusion:**

The prevalence of peripheral arterial disease among hypertensive patients was substantial, and it is associated with history of cigarette smoking, suboptimal blood pressure control, positive family history of the disease, and subjective complaint of leg pain. Thus, it is necessary to activate screening programs for peripheral arterial disease among hypertensive patients, with emphasis on those with predisposing factors.

**Clinical trial number:**

Not applicable.

**Supplementary Information:**

The online version contains supplementary material available at 10.1186/s12872-025-05451-8.

## Introduction

Peripheral artery disease (PAD) is a clinical condition characterized by impaired blood supply to any of the extremities [1]. While PAD can result from a variety of clinical entities causing structural and/or functional occlusion of blood flow, atherosclerosis disease is the most frequent etiology recognized globally [1, 2]. One of the leading risk factors for the development of vascular diseases such as PAD is hypertension, owing to its atherogenic propensity [2, 3].

Hypertension is the leading cardiovascular disease and is the most important risk factor for other cerebrovascular diseases all over the world and in Ethiopia [4, 5]. Globally, 1.4 billion people are estimated to have a high blood pressure [6], with the greatest burden of disease due to hypertension and the largest portion of sub-optimally treated and controlled hypertension being in low- and middle-income countries [7]. Its prevalence varied widely across different regions of the world as a result of multiple interwoven factors [5]. In Ethiopia, a significant proportion of adults, ranging from 35.2% to 38.9%, are estimated to be affected by hypertension, making it a local health concern as well [8, 9].

Previous studies showed that the overall prevalence of PAD among adults with abnormally elevated blood pressure was not uniform, ranging from 8.7% in China [10] to 24.8% in Nigeria [11]. A disproportionately high number of people with PAD live in LMICs, where there is a scarce health resource and limited access to quality health [3]. The corresponding prevalence was 42.6% among elderly Angolan patients [12]. Likewise, the prevalence of subclinical PAD was 10.8% in adults dwelling in Southwest Ethiopia, with hypertension being an independent factor [13]. Epidemiologic data demonstrated an increased risk of PAD among adults with abnormally elevated blood pressure, particularly on the background of factors such as smoking, advancing age, diabetes and hyperlipidemia and black race [10, 11, 14, 15]. As an indicator of generalized atherosclerosis, PAD carries an important risk for cardiovascular morbidity and mortality and decreased quality of life [16–18].

Given its asymptomatic nature [1], the morbidity and mortality attributed to PAD are speculated to be grave in such settings [19] besides of lowering of quality of life among affected ones [16]. Despite the limited empirical data on PAD in sub-Saharan Africa (SSA), the existing literature shows a markedly varying prevalence, with higher prevalence documented in studies that use Doppler based means of diagnosis as opposed to mere clinical evaluation [20]. Despite this varied prevalence and differential burden, screening practice for PAD among high-risk adult patients in SSA such as Ethiopia is poor [21, 22]. Such erratic and suboptimal screening practices of patients with significant CAD are commonly observed in the current study setting.

Owing to the changing patterns of cardiovascular diseases as a result of several non-adaptive lifestyle factors [5], the burden of the disease can be estimated to be high in developing countries such as Ethiopia. In addition to narrowing the knowledge gap, exploring the context-specific magnitude of PAD among such high-risk population is critical in the formulation of successful screening strategies to mitigate its adverse short-term and long-term complications such as coronary heart disease, stroke and foot gangrene [23].

Although several epidemiological studies have been published on PAD recently, data from low resource countries such as Ethiopia are scarce, limiting the amount of evidence for future interventions [17]. Hence, to the authors’ knowledge, this represents the first Doppler-based ankle-brachial index (ABI) assessment specifically targeting hypertensive patients attending tertiary hypertension clinics in Ethiopia, distinguishing it from prior community-based studies in general Ethiopian adult populations [13]. This focused approach addresses a critical evidence gap in high-risk clinical settings.

## Methods and materials

### Study design and settings

An institution-based, cross-sectional study was conducted between March 1st and April 30th, 2023 at Tikur Anbessa Specialized Hospital (TASH) and St. Paul’s Hospital Millennium Medical College (SPHMMC), both located in Addis Ababa, Ethiopia. TASH is the largest and oldest public hospital in the country, providing high-level clinical care for millions of people and offering training for health science students in various disciplines. It has 200 physicians, 379 nurses, and 115 other health professionals dedicated to providing healthcare services. SPHMMC, built in 1969, serves about 200,000 patients annually and has a catchment population of over 5 million people. It is a teaching center for undergraduate and postgraduate health science students and delivers clinical service in various activities, including cardiovascular care.

Both hospitals have three well-functioning hypertension outpatient departments (OPDs), providing clinical service for more than 2460 adult patients with varying follow-up intervals. The service is delivered by nephrologists, internal medicine residents, and trained nurses stationed at each dedicated office.

### Population and sampling

#### Population 

This study’s source population was all patients having regular follow-up at Hypertension clinics of TASH and SPHMMC. Likewise, hypertensive patients selected via convenience sampling technique and who had regular follow-up at Hypertension clinics of TASH and SPHMMC during the study period constituted the study population. Patients were eligible to participate in the study if they were aged eighteen [18] years or more, diagnosed with hypertension, and gave consent to participate in the study. On the contrary, patients with emergency clinical conditions such as advanced heart failure or unstable hemodynamic conditions such as hypertensive crisis, patients with severe leg pain due to leg ischemia or major limb trauma, acute deep vein thrombosis, and prominent peripheral oedema were excluded.

### Sample size determination

The required sample size was determined using Cochran’s formula: $\:\:n=\frac{{z}^{2}p(1-p)}{{e}^{2}}$, assuming the proportion of patients with hypertension having PAD to be 50% due to the absence of previous study done in an Ethiopian setting and with the intention of obtaining maximum sample size, reliability coefficient (z score of 1.96), 95% confidence level, and 5% margin of error. We further adjusted for a 10% nonresponse resulting in a minimum sample size of 422, of which n1 (for TASH) = 225 and n2 (for SPHMMC) = 197.

Random sampling was not feasible owing to clinic constraints: single-shift staffing (2 nurses/session), highly variable attendance (65–85 patients/day), and ABI protocol duration (15–20 min/patient), which risked delaying care and causing discomfort. Systematic approaches were impractical amid unpredictable flow. Convenience sampling was therefore employed among available hypertensive patients at two tertiary referral hospitals specializing in complex cases, following stratification by site.

### Data collection tools and procedures

Data were gathered via interviewer-administered, structured, pre-tested, locally-translated questionnaire. The data collection tool contained socio-demographic characteristics, behavioural variables, clinical variables, and it was adopted from previous similar studies [2, 11, 13]. Intermittent claudication was assessed using the a single claudication question, which was shown to have comparable sensitivity and specificity with the more complex claudication questionnaires, including the Edinburgh Questionnaire [24]. Additionally, medical charts were reviewed whenever it was needed for clinical details.

The ABI measurements were performed by four trained operators who received one-day training by a cardiologist utilizing audiovisual materials to ensure standardization. Each vascular examination was conducted with the patient in the supine position after at least 10 min of rest. Systolic blood pressure (SBP) was measured in the brachial artery of each upper limb and at the dorsal or posterior tibial arteries of the lower limbs using an 8 MHz hand-held vascular Doppler (Summit Doppler LifeDop 250, USA). The ankle-brachial index (ABI) for each limb was calculated by dividing the SBP at the ankle by the higher SBP measured in the arms.

ABI values greater than 1.40, indicative of medial arterial calcification commonly seen in diabetic and hypertensive populations, were excluded to avoid misclassification. Only a single measurement per limb was recorded during each examination. Inter- and intra-observer variability were not assessed in this study. The handheld Doppler ultrasound device used demonstrated high sensitivity, specificity, and diagnostic accuracy of 88.89%, 90.70%, and 89.57%, respectively [25].

### Study variables and operational definitions

The dependent variable for this study was peripheral artery disease. Hypertension was diagnosed in the cohort by treating physicians or by current use of prescribed antihypertensive agents. Peripheral artery disease was operationalized as a pathological hypoperfusion to the lower extremity/ies as evidenced by ABI ≤ 0.90 using Doppler ultrasound [11, 13, 25, 26]. Current smoker represented an active smoker or a smoker who quitted within the previous one 1 year while former smoker included a smoker who had quitted smoking for more than a year before the time of interview [27, 28]. Non-sedentary lifestyle embodied subjects doing activity involving physical effort for at least 30 min a day for a minimum of 5 days a week (household activities involving physical effort, walking to and from work involving at least 30 min, manual workers, those performing leisure-time physical activities. All others were classified as sedentary [29].

### Data processing and analysis

Data entry, coding and cleaning were performed using Epi-info version 7.2.2.2, and statistical analysis was done using Statistical Package for Social Science (SPSS) version 26. Frequency and cross tabulation were used to check for missed values and variables. The demographic and clinical characteristics of participants were computed using descriptive statistics such as median + interquartile range based on the distribution of data along with frequencies and percentages.

Following Babyak and Event Per Variable (EPV) guidelines [30, 31], the final logistic regression model was limited to four predictors (32 EPV) to mitigate overfitting, as indicated by an initial Hosmer-Lemeshow p-value < 0.05. Parsimonious models including 4 variables were preferred over automated selection methods. Sensitivity analyses reduced the Hosmer-Lemeshow p-value from 0.002 to 0.075, indicating improved model fit.

Potential multicollinearity among independent variables was checked prior to multivariable analysis using Variance Inflation Factor (VIF) and tolerance statistics. All variables met acceptable thresholds (VIF < 2.5; tolerance > 0.4), indicating no significant multicollinearity. There were no missing data, as information was obtained from both patient interviews and medical records.

Finally, binary and multiple logistic regression analyses were performed to determine the potential factors associated with the outcome variable. Clinically pertinent variables with a p-value < 0.25 in bivariable logistic regression screening were retained in the multiple regression model. The strength association was computed using adjusted odds ratios with 95% confidence intervals. A p-value was considered significant at the level of < 0.05.

## Results

### Sociodemographic characteristics of patients

Out of the total 422 hypertensive patients approached for data collection, complete data were obtained for 408 patients, yielding a response rate of 96.7%. Females accounted for well more than half (232; 56.9%) of the study population, with a female to male ratio of 1.3:1. Patients’ age ranged from 30 to 84 years, with median (interquartile range) of 60 (54–67). Further, most (347; 85%) of the studied patients reported to be urban dwellers (Table 1).

**Table 1:** Background characteristics of patients having regular follow-up at hypertension clinics of TASH and SPHMMC, addis Ababa, Ethiopia from March 1st to April 30th, 2023 (*n* = 408)

Variable	Frequency	Percent (%)
Age strata		
< 45 years	32	7.8
45–54 years	75	18.4
55–64 years	165	40.4
≥ 65 years	134	33.3
Sex		
Male	176	43.1
Female	232	56.9
Residence		
Urban	347	85.0
Rural	61	15.0
Religion		
Orthodox	261	64.0
Muslim	82	20.1
Protestant	47	11.5
Catholic	18	4.4
Current marital status		
Unmarried	20	4.9
Married	253	62.0
Divorced/separated	42	10.3
Widowed	93	22.3
Educational status		
No formal education^€^	101	24.7
Primary education	84	20.6
Secondary education	130	31.9
College diploma and above	93	22.8
Occupational category		
Housewife	128	31.4
Unemployed	101	24.8
Merchant	76	18.6
Employee	43	10.5
Retired	41	10.0
Others	19	4.7
Household monthly income quintile*		
Lowest (≤ 3000)	132	32.4
Lower (3001–3500)	49	12.0
Middle (3501–5000)	110	27.0
Higher (5001–6000)	55	13.5
Highest (> 6000)	62	15.2

^€^ Can read/write = 12

^*^ Income is expressed in Ethiopian Birr and was categorized in quintiles

In terms of religious affiliation, about two-thirds of the study population were orthodox Christians (*n* = 261), and a fifth (*n* = 82) were Muslims. Nearly two-thirds were married (*n* = 253), and more than a fifth (93; 22.3%) were widowed. Approximately a third (130; 31.9%) attended up to secondary education whereas about a quarter (*n* = 101) had no formal education. More than half (229; 56.2%) reported to be either housewives or unemployed. With respect to family income, close to one-third (32.4%; 132) of the patients stated to have an average monthly income less than 3000 Ethiopian Birr (Table 1).

### Behavioural and clinical characteristics of patients

Most 373 (91.4%) of the patients reported no history of cigarette smoking, with only thirty-five (8.6%) having a history of smoking. Out of those who smoked, most (*n* = 30; 85.7%) were active smokers. Likewise, only forty-four (10.8%) described to consume alcoholic beverages whereas seven (1.7%) stated to chew chat. Nearly one-third of all patients (*n* = 124; 30.4%) reported to do regular physical exercise. Besides, majority (46.6%; *n* = 190) of the patients had normal BMI measurements (18.5–24.9) while a hundred forty-seven (36%) were obese as they had a BMI measurement ranging from 25 to 29.9 (Table 2).

**Table 2:** Clinical and behavioral characteristics of patients having regular follow-up at hypertension clinics of TASH and SPHMMC, addis Ababa, Ethiopia from March 1st to April 30th, 2023 (*n* = 408)

Variable	Frequency	Percent (%)
Cigarette smoking		
Yes	35	8.6
No	373	91.4
Type of smoking (*n* = 35)		
Active	30	85.7
Former	5	14.3
Alcohol consumption		
Yes	44	10.8
No	364	89.2
Chat chewing		
Yes	7	1.7
No	401	98.3
Regular physical exercise		
No	284	69.6
Yes	124	30.4
Body mass index		
< 18.5	16	3.9
18.5–24.9	190	46.6
25–29.9	147	36.0
≥ 30	55	13.5
Duration		
< 5 years	111	27.2
5–10 years	160	39.2
> 10 years	137	33.6
Number of anti-hypertensive drugs		
1	123	30.1
≥ 2	285	69.9
Presence of comorbidity		
No	137	33.6
Yes	271	66.4
BP control		
Suboptimal	251	61.5
Optimal	157	38.5
Family history of PAD		
Yes	21	5.1
No	387	94.9
Leg pain		
Yes	137	33.6
No	271	66.4

*BP* Blood pressure, *PAD* Peripheral arterial disease

**Fig. 1:**
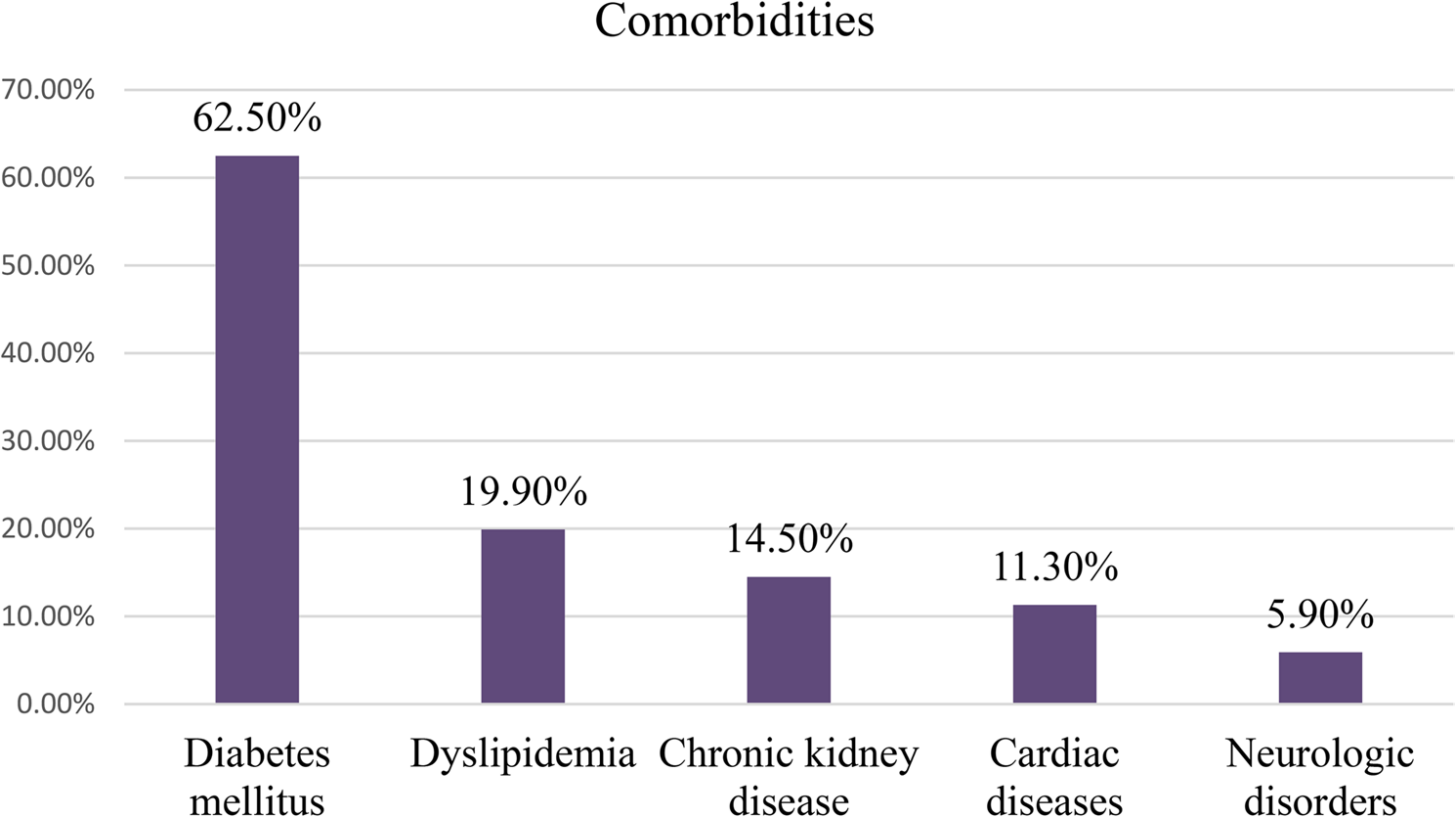
Frequency of comorbidities among patients having regular follow-up at Hypertension clinics of TASH and SPHMMC, Addis Ababa, Ethiopia from March 1st to April 30th, 2023 (*n* = 408)

Majority 160 (39.2%) of patients had been hypertensive for 5 to 10 years prior to the data collection period. Most (*n* = 285; 69.9%) of the patients were on polytherapy, taking at least two blood pressure lowering agents. Two-thirds of the studied patients (*n* = 271; 66.4%) had a chronic comorbid medical condition, with diabetes mellitus (*n* = 255; 62.5%) and dyslipidemia (*n* = 81; 19.9%) being the most commonly identified comorbidities (Fig. 1). In addition to this, majority had suboptimal BP control (61.5%; *n* = 251). Further, only twenty-one (5.1%) patients reported a positive family history of PAD, and one-third (*n* = 137; 33.6%) complained of leg pain (Table 2).

### Prevalence of PAD

**Fig. 2:**
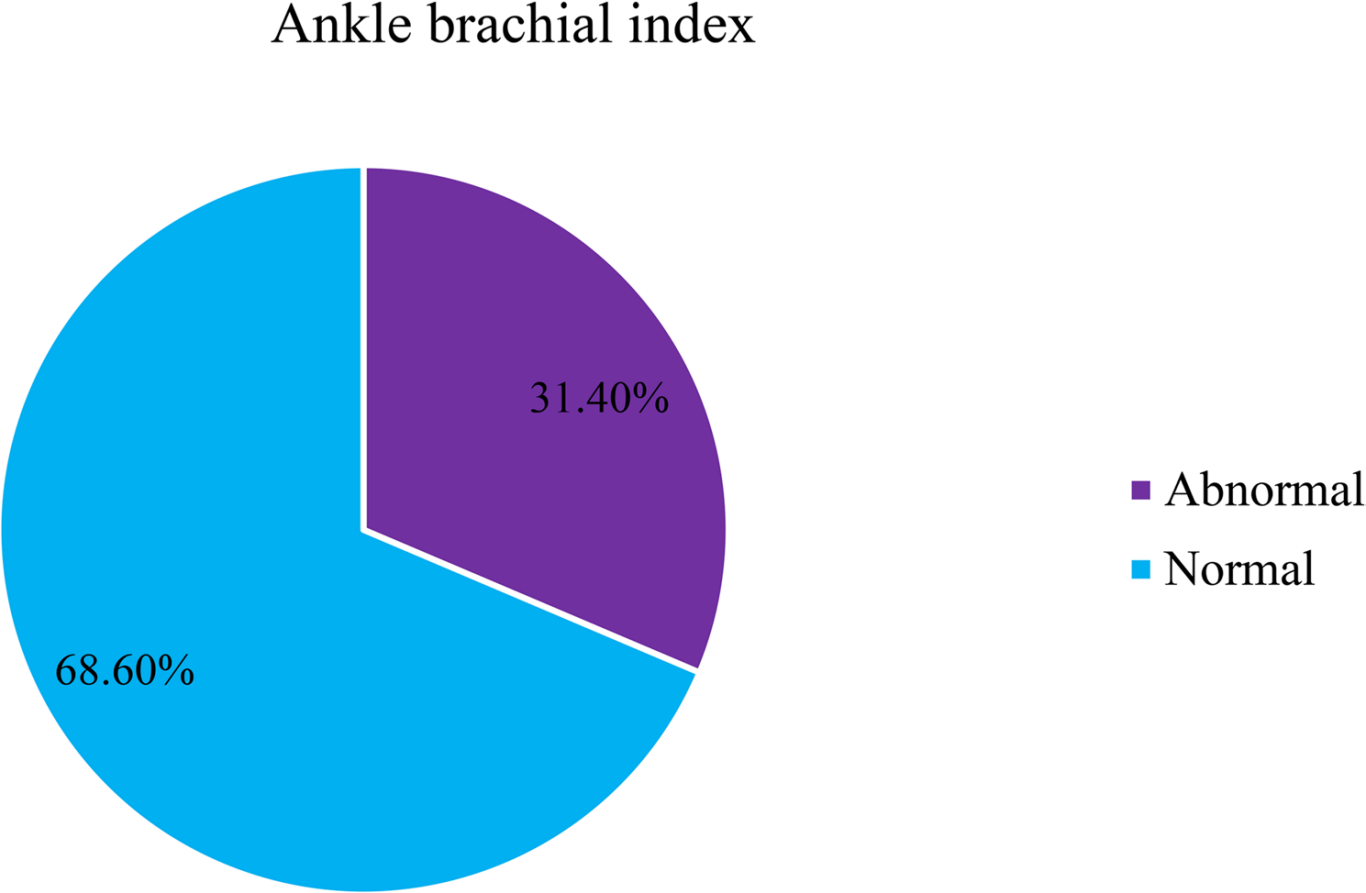
Ankle brachial index profile among patients having regular follow-up at Hypertension clinics of TASH and SPHMMC, Addis Ababa, Ethiopia from March 1st to April 30th, 2023 (*n* = 408)

In the overall sample, the prevalence of PAD was 31.4% (95%CI:26.8%–35.9%) as ABI was found to be ≤ 0.90 PAD among 128 of the patients (Fig. 2).

### Factors associated with PAD

This study showed that, when compared to patients who stated not to have history of cigarette smoking, those who reported to have any history of smoking were more likely [AOR = 9.99 (95%CI: (4.22,23.66)] to have abnormal ABI. Similarly, those with suboptimal blood pressure control in their latest follow-up had about three-fold likelihood of having abnormal ABI [AOR(95%CI): 2.73(1.65,4.51)] in comparison to their counterparts with optimal control. Moreover, in reference to patients with no family history of PAD, patients with family history of PAD had nearly four times odds of having abnormal ABI [AOR(95%CI) = 3.59(1.37,9.42)], Finally, patients with history of leg pain were more likely to have abnormal ABI [AOR = 1.98 (95%CI: 1.23,3.17)] in reference to patients who had no leg pain as a complaint (Table 3).

**Table 3:** Factors associated with ankle brachial index among patients having regular follow-up at hypertension clinics of TASH and SPHMMC, addis Ababa, Ethiopia

Variable	Ankle brachial index		COR(95%CI)	AOR(95%CI)	*P* value
	Abnormal (%)	Normal (%)			
Cigarette smoking					
Yes	27(77.1)	8(22.9)	9.09(4.00,20.66)	9.99(4.22,23.66)	**< 0.001**
No	101(27.1)	272(72.9)	1	1	-
BP control					
Suboptimal	98(39.0)	153(61.0)	2.71(1.69,4.35)	2.73(1.65,4.51)	**< 0.001**
Optimal	30(19.1)	127(80.9)	1	1	-
Family history of PAD					
Yes	12(57.1)	9(42.9)	3.12(1.28,7.59)	3.59(1.37,9.42)	**0.009**
No	116(30.0)	271(70.0)	1	1	-
Leg pain					
Yes	55(40.1)	82(59.9)	1.82(1.18,2.81)	1.98(1.23,3.17)	**0.005**
No	73(26.9)	198(73.1)	1	1	-

Only variables with *p* value < 0.25 in binary logistic regression are shown here

*BP* Blood pressure, *PAD* Peripheral arterial disease

1: reference category

## Discussion

This study aimed to assess the prevalence of peripheral artery disease and its associated factors among patients with hypertension attending two tertiary hospitals in the context of Addis Ababa, Ethiopia. The results demonstrated that about one-third of the hypertensive patients had deranged ABI measurements, with certain clinical factors such as history of cigarette smoking, suboptimal BP control, positive family history of PAD, and subjective complaint of leg pain contributing to presence of PAD.

Approximately one-third of the hypertensive patients had PAD according to ABI measurements. The prevalence of PAD in the current study was higher than the result of the study done in Nigeria, which documented a prevalence of 11% among hypertensive patients [2]. Similarly, it was higher than the prevalence of 8.7% obtained among Chinese patients with hypertension [10]. The present figure was also comparatively higher than the observation Hungarian context wherein a prevalence of PAD (ABI ≤ 0.9) was found to be 14.4% among hypertensive patients [32]. On the contrary, the present prevalence of PAD was lower than the one documented among Angolan population (42.6%) [12]. Again, the current finding was quite different from the observation (41.8%) documented among hypertensive patients at Benin city, Nigeria [33].

The plausible explanations for the observed high prevalence of PAD in this study include high prevalence of pre-existing diabetes mellitus (62.5%), overweight (36%) and obesity (13.5%) and overweight (26.7%), and suboptimal blood pressure control and lipid control among the study population.

One of the possible reasons for the differences might be variations in the type of diagnostic tool used. The current study used the ankle-brachial index as its diagnostic methods, which can underestimate the prevalence of PAD, while others used color Doppler ultrasound or automated oscillometric method. Moreover, such discrepancy can be explained by the variation in demographic and clinical characteristics of study subjects, sample size, and study design.

Further, the higher prevalence of PAD observed in the current study in comparison with that of the Chinese and Hungarian study can be justified by the notion that PAD is more common among black population than their white counterparts [11, 33–35]. Additionally, the relatively elevated PAD prevalence may reflect the older age distribution in this cohort (73.7% aged ≥ 55 years), a substantial comorbidity burden (62.5% with diabetes, 19.9% with dyslipidemia), and tertiary-care referral patterns that concentrate patients with advanced disease. This contrasts with population-based surveys where hypertension and diabetes mellitus prevalence were 29.5% and 6.8%, respectively, and the majority (46.6%) were aged 50 years or younger [13]. Given that this study was conducted in tertiary hypertension clinics, the sample likely includes patients with more complex disease profiles, potentially accentuating the burden of PAD in the broader community and underscoring the need for validation in primary care settings.

The current study confirmed that the odds of PAD were significantly higher in cigarette smokers than non-smokers. This finding is aligned with that of several other studies done previously which stressed the strong association between tobacco smoking and PAD [28, 36]. The observed association aligns with established pathophysiological mechanisms, whereby nicotine, carbon monoxide, and other tobacco constituents exert toxic effects on blood vessels. These toxins stimulate catecholamine release and activate endogenous free radicals, promoting oxidative stress, platelet aggregation, and thrombus formation [37, 38]. However, the cross-sectional design precludes causal inference.

Another notable finding of this study was that suboptimal BP control was associated with the development of PAD. This is supported by an earlier American study, who reported that PAD patients displayed a J-shape relationship with systolic blood pressure, suggesting a lower target blood pressure among the patients [39]. It was also in line with the finding of the aforementioned Hungarian report, which stated that the prevalence of PAD was significantly lower in hypertensive patients achieving their target blood pressure [32].

Moreover, positive family history of PAD increased the odds of PAD in this study, and this is in agreement with the population-based cohort study, namely The San Diego Population Study (SDPS), which highlighted that a positive history of PAD in first degree relative was a strong factor for PAD [40]. Finally, the present study revealed that subjective complaint of leg pain was associated with deranged ABI measurements. This finding is particularly supported by a previous study, which documented that claudication (leg pain on walking) is significantly increases the likelihood of having a reduced ABI [24].

To the authors’ knowledge, this preliminary study represents the first Doppler-based ABI assessment of PAD prevalence and associated factors among hypertensive patients attending tertiary care clinics in Ethiopia. Several limitations warrant acknowledgment. First, convenience sampling from tertiary referral centers introduced selection bias by overrepresenting complex cases, thereby limiting generalizability to broader hypertensive populations in Addis Ababa or Ethiopia. Second, the cross-sectional design precludes inferences on temporality or causality between examined factors and PAD. Third, reliance on single-timepoint ABI for PAD diagnosis risks misclassification bias due to intra-individual measurement variability; moreover, ABI may underestimate PAD prevalence in diabetics (62.5% of cohort) owing to medial arterial calcification or in elderly patients (73.7% aged ≥ 55 years) due to vessel incompressibility, while potentially overestimating it in non-affected individuals. Fourth, smoking classification did not account for pack-years or cumulative exposure, which might affect the strength of the observed association. In a similar vein, potential recall bias in self-reported smoking and exercise habits may further influence exposure measurement accuracy. the high prevalence of coexisting conditions such as diabetes (62.5%), dyslipidemia (19.9%), and uncontrolled hypertension (61.5%) may represent unmeasured confounders due to incomplete adjustment for disease severity.

## Conclusion and recommendation

The prevalence of PAD among hypertensive patients was substantial compared to regional hypertensive or general adult cohorts, affecting one in three patients. History of cigarette smoking, suboptimal BP control, positive family history of PAD, and subjective complaint of leg pain were significantly associated with the presence of PAD. These findings underscore the need for clinicians to consider routine PAD screening using noninvasive techniques like ABI among hypertensive patients. Local healthcare authorities should strengthen educational programs for clinical practitioners to encourage PAD screening. Future researchers should conduct large-scale studies to validate these findings with prospective longitudinal designs for better outcomes.

## Supplementary Information

Supplementary Material 1.

## Data Availability

All data in which the conclusions of the study were based are available. The data that support the findings of this study are available from the corresponding author upon reasonable request, with permission of each study institution.

## Abbreviations

ABI: Ankle Brachial Index
AOR: Adjusted Odds Ratio
BMI: Body Mass Index
BP: Blood Pressure
COR: Crude Odds Ratio
HTN: Hypertension
PAD: Peripheral Arterial Disease
SPHMMC: St. Paul’s Hospital Millennium Medical College
SPSS: Statistical Package for Social Sciences
SSA: Sub-Saharan Africa
TASH: Tikur Anbessa Specialized Hospital
VIF: Variance Inflation Factor
