# Supplementary material for: Prevalence of peripheral arterial disease and associated factors among hypertensive patients attending two tertiary hospitals of Addis Ababa, Ethiopia
Source: BMC Cardiovasc Disord. 2025 Dec 18;26:68. doi: 10.1186/s12872-025-05451-8 (PMC12825174; doi:10.1186/s12872-025-05451-8)
Supplement: Supplementary file 1 — Supplementary Material 1. [file 12872_2025_5451_MOESM1_ESM.docx]

# Annex

# Annex I: English version questionnaire

**St. Paul’s Hospital Millennium Medical College**

**Department of Medical-Surgical Nursing**

**Participant Information Sheet and Informed Consent Form**

Hello dear respondents, my name is_____________________________. I am here to collect data for the research purpose which is conducted to complete a thesis for master’s degree in Cardiovascular Nursing at SPHMMC.

The purpose of this study is to assess the prevalence of peripheral artery disease and its associated factors among patients with hypertension attending two tertiary hospitals of Addis Ababa, Ethiopia

You are selected to be one of the participants for this study. I would like to ask you to fill this questionnaire that takes 10 to 15 minutes of your time. No harm is imposed to you except the time you commit for interview but some of the question may look too personal but it is helpful for the study. In addition, there is no payment for participation even though the result of the study may benefit as a citizen. The participation in this study is entirely voluntary, you have the right to refuse or withdraw from the study at any time for any reason without penalty. However, your honest answers to these questions are important since it provide relevant information to design interventions that aims to improve the quality of life of cardiac patients

The information you provide is confidential and it will be used only for study purpose and it will not be disclosed to anyone. A code number will be used to identify the participant therefore, writing your name is not needed.

If you have something that is not clear about the study please contact the principal investigator, Mrs. Semira Khairdin Jemal (Mobile phone: +251911553711, email: semirakhair.55@gmail.com) at any time.

Are you willing to participate in this study? 1. Yes 2. No

Signature of data collector certifying verbal informed consent____________

Thank You!!!

**English version questionnaire**

**St. Paul’s Hospital Millennium Medical College**

**Department of Medical-Surgical Nursing**

This is the questionnaire that will be used to assess the prevalence of peripheral artery disease and its associated factors among patients with hypertension attending two tertiary hospitals of Addis Ababa, Ethiopia

001. Data collector: code ____/___/___ Name ______________________

002. Date of data collection ____/___/____ Time ___________

003. Checked by Supervisor: Signature ________day _______month ______ year____

101. Serial number______________ 102. Card number______________

**Part I. Socio-demographic data of the patient**

| **S. N** | **Questions** | **Response category** | **Skip** |
| --- | --- | --- | --- |
| 101 | Age in years | ___________________ |  |
| 102 | Sex | 1. Male 2. Female |  |
| 103 | Residence | 1. Urban 2. Rural |  |
| 104 | Marital status | 1. Single 2. Married 3. Divorced/separated 4. Widowed |  |
| 105 | Educational level | 1. Not attended formal education 2. Can read/write 3. Primary education 4. Secondary education 5. Higher level education (College/University) |  |
| 106 | Your current occupation | 1. Daily laborer 2. Farmer 3. Merchant 4. Private work 5. Governmental employee 6. Other (specify_______________) |  |
| 107 | Religion | 1. Orthodox 2. Muslim 3. Protestant 4. Catholic 5. Other (specify……..) |  |
| 108 | Average monthly household income in Ethiopian Birr | _________________ |  |
| **Part 2. Behavioral and related variables** | | |  |
| 201 | Do you smoke cigarette? | 1. Yes 2. No |  |
| 202 | If your answer to question number 201 is “1”, specify: | 1. Former smoker 2. Active smoker | Skip if answer question number 201 is “2” |
| 203 | Do you take alcohol? | 1. Yes 2. No |  |
| 204 | Do you chew chat? | 1. Yes 2. No |  |
| 205 | Do you use any psychoactive substance? | 1. Yes 2. No |  |
| 206 | Do you do any activity involving physical effort for at least 30 minutes a day for a minimum of 5 days a week? | 1. Yes 2. No |  |
| 207 | Height in meter | __________________ |  |
| 208 | Weight in kilograms | __________________ |  |

**Part III. Clinical data**

| **S. N** | **Questions** | **Response category** | **Skip** |
| --- | --- | --- | --- |
| 301 | The time duration since the diagnosis of hypertension was made? | ______year(s)_________months |  |
| 302 | Age at the diagnosis of hypertension | ___________________ |  |
| 303 | How many BP lowering medications do you take? | __________________ |  |
| 304 | What antihypertensive medication(s) the patient is taking | 1. ACE inhibitors 2. Beta blockers 3. Calcium channel blockers 4. Diuretics |  |
| 305 | What was your last BP record (mmHg)? |  |  |
| 306 | Do you have any comorbidity? | - 1. Yes   2. No |  |
| 307 | If your answer to question number ‘306’ is “1”, specify the type of comorbid condition | 1. Diabetes mellitus 2. Dyslipidemia 3. Chronic kidney disease 4. HIV/AIDS 5. Stroke 6. Cardiac disease 7. Chronic liver disease 8. Chronic lung disease 9. Other (specify________) | Skip this item if the answer to Q306 is “2” |
| 308 | Do you have family history of PAD? | 1. Yes 2. No |  |
| 309 | Do you get any pain or discomfort in your legs during normal walking? | 1. Yes 2. No |  |
| 310 | Tibial posterior artery systolic pressure | __________________ |  |
| 311 | Tibial anterior artery systolic pressure | ________________ |  |
| 312 | Highest arm systolic pressure | _________________ |  |
| 313 | Ankle brachial index | __________________ |  |

**Thank the respondent!**
